# Development and 2025 re-evaluation of a Japanese quality indicator set for adult intensive care: a modified RAND/UCLA Delphi study

**DOI:** 10.1186/s40560-026-00863-w

**Published:** 2026-02-10

**Authors:** Junji Kumasawa, Hiroshi Okamoto, Takaki Naito, Takehiko Asaga, Masayoshi Kimura, Toru Kotani, Yasuhiro Komatsu, Takeshi Suzuki, Tetsuya Takahashi, Tetsuhiro Takei, Masaji Nishimura, Satoru Hashimoto, Hiroko Yamaguchi, Matsuyuki Doi

**Affiliations:** 1 Department of Critical Care Medicine, Sakai City Medical Cente, 1-1-1 Ebaraji-Cho, Nishi-Ku, Sakai, Osaka 593-8304 Japan; 2 Kyoto University Graduate School of Medicine, Human Health Science, Kyoto, Japan; 3 Department of Critical Care Medicine, Luke’s International Hospital, Tokyo, Japan; 4 Department of Emergency and Critical Care Medicine, St. Marianna University School of Medicine, Kanagawa, Japan; 5 Intensive Care Unit, Kagawa University Hospital, Kagawa, Japan; 6 Department of Clinical Engineering, Hyogo Medical University Hospital, Hyogo, Japan; 7 Department of Intensive Care Medicine, Showa Medical University, Tokyo, Japan; 8 Patient Safety Education Center for Multiprofessionals, Gunma University, Gunma, Japan; 9 Itabashi Chuo Medical Center, Tokyo, Japan; 10 Department of Anesthesiology, Tokai University School of Medicine, Kanagawa, Japan; 11 Faculty of Health Science, Juntendo University, Tokyo, Japan; 12 Department of Emergency and Critical Care Medicine, Yokohama City Minato Red Cross Hospital, Kanagawa, Japan; 13 Aizenbashi Hospital, Osaka, Japan; 14 Nonprofit Organization Intensive Care Collaboration Network, Tokyo, Japan; 15 Nagoya Ekisaikai Hospital, Aichi, Japan; 16 Department of Intensive Care, Hamamatsu Medical Center, Shizuoka, Japan

**Keywords:** Quality of care, Quality indicators, Intensive Care Unit, RAND, Delphi method, Administrative claims data

## Abstract

**Background:**

Quality indicators (QIs) support measurement and improvement of ICU care; however, until 2017 there was no comprehensive, evidence-based QI set tailored to frontline adult ICU practice in Japan. Because evidence and practice evolve, periodic updating is required. We developed a Japanese ICU QI set in 2017–2018 and conducted a formal re-evaluation in 2025 to confirm current validity and update the core set.

**Methods:**

We used a modified RAND/UCLA appropriateness method. First, an initial list of candidate QIs was generated through a systematic literature review and major clinical guidelines. Next, a multidisciplinary panel of 14 Japanese experts (including intensivists, a nurse, a physiotherapist, and a clinical engineer) rated the appropriateness of these indicators using a nine-point Likert scale (Round 1). Following Round 1, a face-to-face consensus meeting was held to discuss, refine, add, or delete indicators based on scientific evidence, clinical importance, and feasibility, followed by Round 2 to finalize the 2018 consensus set. In 2025 (Round 3), the panel re-rated all indicators from the 2018 set using the same rating and classification framework and also rated newly proposed indicators reflecting contemporary practice.

**Results:**

The systematic review yielded 44 initial candidate QIs. After the two-round rating process and the expert panel meeting, a 2018 consensus set of 38 QIs was established (13 structure indicators, 10 process indicators, and 15 outcome indicators). In Round 3 (2025), 37 indicators remained Appropriate, whereas one indicator was reclassified as Uncertain and was not retained in the updated core set. One newly proposed indicator was rated Appropriate and added, resulting in an updated 2025 core set of 38 indicators. All selected indicators were deemed appropriate and relevant for the Japanese ICU setting by the expert panel.

**Conclusions:**

Using a rigorous modified RAND/UCLA appropriateness method and a 2025 re-evaluation, we developed and updated a feasible, contextually adapted Japanese ICU QI set. The updated core set, with operational definitions and specified data sources, provides a foundation for national benchmarking and continuous quality improvement in Japanese ICUs.

330 words.

**Supplementary Information:**

The online version contains supplementary material available at 10.1186/s40560-026-00863-w.

## Introduction

Intensive care units (ICUs) provide life-sustaining care for the most critically ill patients, but this care is complex, resource-intensive, and carries inherent risks. Ensuring the delivery of consistent, high-quality care is, therefore, a global priority to optimize patient outcomes and improve healthcare efficiency. Quality indicators (QIs) have emerged as a standard, evidence-based tool for measuring, monitoring, and improving the quality of healthcare across various disciplines. Rooted in Donabedian’s classic framework of structure, process, and outcome [1], QIs translate clinical evidence into measurable metrics that can reveal gaps between recommended and actual practice, thereby identifying opportunities for improvement.

In the field of critical care, several international bodies, such as the European Society of Intensive Care Medicine (ESICM), have developed foundational QI sets that have advanced the science of quality measurement [2]. These international efforts provide an invaluable framework and a common language for discussing ICU quality. However, the direct application of these indicator sets across different national healthcare systems can be challenging. Significant variations in clinical practice patterns, patient demographics, data infrastructure, and resource availability can limit the relevance and feasibility of indicators developed in one context when applied to another.

This issue has been particularly pertinent in Japan. Although Japan has a sophisticated healthcare system and a national ICU patient registry (the Japanese Intensive care PAtient Database, JIPAD) [3], until we initiated this study in 2017, there had been no comprehensive, evidence-based set of QIs developed specifically to assess the quality of frontline clinical care in Japanese ICUs. This absence of a standardized, contextually validated toolkit was a significant barrier to nationwide quality benchmarking, identifying areas for improvement, and systematically evaluating the impact of quality improvement initiatives. A dedicated QI set tailored to the Japanese healthcare environment was, therefore, urgently needed. To address this gap, we developed a Japanese ICU QI set through a formal multidisciplinary consensus process conducted in 2017–2018 using a modified RAND/UCLA appropriateness method.

However, quality indicator sets are not static. Because critical care evidence and clinical practice evolve rapidly, indicator sets require periodic re-evaluation and updating to maintain current validity and feasibility. To ensure that this QI set remains relevant to contemporary practice, we, therefore, performed an additional re-evaluation in 2025 using the same RAND/UCLA-based rating approach.

Therefore, this study reports [1] the development of a comprehensive and evidence-based set of quality indicators for adult ICU care tailored to the Japanese healthcare system using a modified RAND/UCLA appropriateness method and a formal multidisciplinary consensus process, and [2] a 2025 re-evaluation round to update and assess the current validity of the resulting indicator set.

## Methods

### Study design

This is a Delphi study to develop and update a set of QIs for the care of patients in ICUs. We used the modified RAND/UCLA appropriateness method to develop a set of QIs by combining expert opinion and scientific evidence [4]. This consensus method has been widely used to develop QIs [5–7]. The initial development phase (2017–2018) comprised four steps. First, we identified a list of candidate indicators through a systematic literature review and relevant clinical guidelines. Second, we carried out a first round of questionnaires, asking members of the expert panel to assess the appropriateness of each potential indicator. Third, an in-person expert panel meeting was held, during which the panelists reviewed and discussed the appropriateness of the indicators based on the results from the first questionnaire round. Fourth, we conducted a second round of questionnaires to finalize the 2018 consensus set of quality indicators. To address the substantial time gap since the original consensus process (2017–2018), we additionally conducted a re-evaluation round in 2025 (Round 3), in which the expert panel re-rated the final 2018 QI set using the same RAND/UCLA rating approach, with an option to rate newly proposed indicators reflecting contemporary practice. This study did not involve analysis of patient-level data from electronic health records; all evaluations were based solely on the expert panel’s ratings.

This study protocol was reviewed and approved by the Ethics Committee of Sakai City Medical Center (Approval No. S-17-061; date: 10 Dec 2017).

### Quality indicators

QIs are crucial instruments for evaluating healthcare quality, providing a scientific basis for evaluating performance. The calculation of QIs typically relies on two primary components:

Denominator: The defined patient population or condition to which the QI applies.

Numerator: The criteria outlining appropriate treatment or desired outcomes.

QI can be utilized to determine either:

The proportion of patients receiving appropriate treatment within a given facility.

The number of indicators achieved at the individual patient level.

Rooted in Donabedian’s framework, which encompasses three dimensions: structure, process, and outcome, process indicators, which describe the delivery or reception of care, are particularly valuable as they represent modifiable factors directly influencing quality improvement [1].

### Step 1: Systematic review and selection of indicator candidates

We systematically searched MEDLINE from Jan 1946 to 6th Nov 2017. The full search strategies are described in the Supplementary Methods 1. Then, we extracted candidate indicators from the full-text review. Three intensive care specialists (JK, HO and TN) assessed and extracted data independently.

### Step 2: First questionnaire round

The expert panel for this study comprised fourteen professionals from diverse medical and allied health disciplines, including ten intensivists, one nephrologist, one physiotherapist, one clinical engineer, and one nurse, selected from different geographical regions and various types of institutions, including both academic and community hospitals, to ensure a broad representation of clinical practice in Japan. We distributed a questionnaire via email, requesting each expert to evaluate the appropriateness of potential quality indicators using a nine-point Likert scale (1 = extremely inappropriate; 9 = extremely appropriate). The nine-point scores were divided into three ranges: 1–3 (inappropriate), 4–6 (uncertain), and 7–9 (appropriate). Each indicator was then classified as “Inappropriate”, “Uncertain,” or “Appropriate” based on the median score and the level of panel agreement/disagreement.

- An indicator was classified as Inappropriate if its median score was in the 1–3 range, without disagreement.
- An indicator was classified as Uncertain if its median score was in the 4–6 range, or if the panel’s ratings showed disagreement, regardless of the median.
- An indicator was classified as Appropriate if its median score was in the 7–9 range, without disagreement.

For the primary analysis, disagreement was defined as the situation in which at least five panelists rated an indicator in the low-appropriateness range [1–3] and at least five panelists rated it in the high-appropriateness range [7–9]. As a sensitivity analysis, we also assessed disagreement using the interpercentile range adjusted for symmetry (IPRAS) method described in the RAND/UCLA manual [8] and confirmed that the overall classification did not change when using the IPRAS-based definition (see Supplementary Methods 2).

Indicators classified as ‘Appropriate’ or ‘Uncertain’ were carried forward for discussion at the subsequent expert panel meeting.

### Step 3: Expert panel meeting

All panel members participated in a face-to-face meeting to review the preliminary results from the initial questionnaire round. The primary purpose of this meeting was to facilitate open discussions on each potential indicator and to reach a consensus. During the meeting, the experts qualitatively evaluated the indicators based on three key criteria: (1) broad applicability across diverse intensive care settings, (2) measurability using data sources commonly available in Japan (e.g., electronic health records, administrative claims, registry data, and, where applicable, facility-level surveys), and (3) relevance to quality improvement, with particular emphasis on actionable (modifiable) process indicators. Indicators failing to meet these criteria were either revised or excluded. In addition, experts were encouraged to propose new potential indicators if deemed necessary. These newly suggested indicators were qualitatively assessed in the meeting and subsequently included in the second quantitative assessment.

In this study, “facility-level surveys” refer to institutional surveys (e.g., staffing, protocols, and safety systems) rather than patient-reported surveys. For hand hygiene, the survey is intended to capture facility-reported compliance results from local audit/monitoring programs based on the WHO direct observation framework, in which compliance is defined as the proportion of fulfilled hand hygiene opportunities among observed opportunities.

### Step 4: Second questionnaire round and final selection (round 2; 2018 consensus set)

Following the expert panel meeting, a revised list of accepted, modified, and newly proposed indicators was compiled into a second questionnaire. This questionnaire was then sent to panel members via email for final evaluation. In this second round, indicators were re-rated using the same nine-point Likert scale as the first round. Indicators achieving “Appropriate” defined above constituted the final 2018 consensus set of quality indicators for care of patients in ICUs [5, 8, 9].

### Step 5: Re-evaluation round (round 3; 2025 update)

In 2025, the expert panel re-rated all indicators from the 2018 consensus set to re-assess their current validity. The Round 3 questionnaire was distributed by email to all 14 members of the original panel, and all 14 panelists returned complete ratings (response rate: 14/14, 100%). The re-evaluation was conducted as an email-based survey without an additional face-to-face or virtual consensus meeting between ratings. Individual ratings were not shared with other panelists during Round3. To support a time-updated appraisal, panelists were provided with the finalized 2018 indicator definitions and the key background materials used in the original process, together with selected updates reflecting contemporary practice where relevant. A free-text comment field was included for each indicator, and panelists were invited to propose additional candidate indicators reflecting evolving standards; newly proposed candidates were compiled and circulated for rating within the same RAND/UCLA-based framework. The same nine-point scale and classification rules were applied. Indicators rated as “Appropriate” in Round 3 were retained in the updated set, whereas indicators rated as “Uncertain” or “Inappropriate” were not retained as core QIs but were reported transparently.

### Statistical analysis

This study did not involve analysis of patient-level data but relied solely on scores rated by the expert panel. We summarized ratings using medians and RAND/UCLA-based agreement/disagreement rules. Disagreement was assessed primarily using the predefined count-based definition and, as a sensitivity analysis, using the IPRAS method. Details of the IPRAS calculation and the comparison between definitions are provided in Supplementary Methods 2.

## Results

**Fig. 1:**
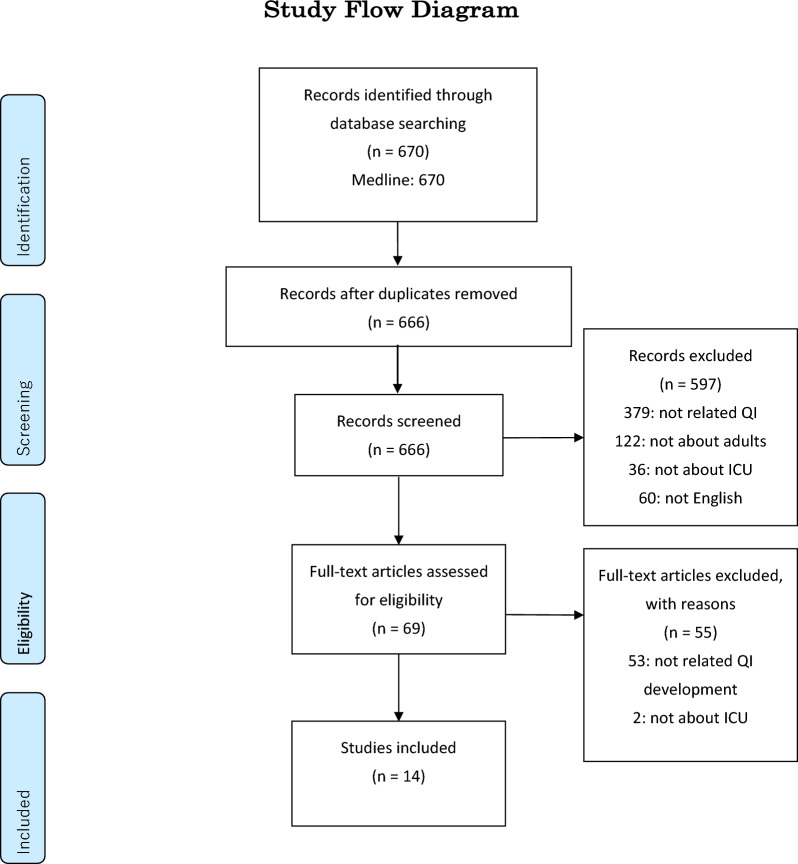
PRISMA flow diagram of the study selection process. The diagram details the process of identifying, screening, and selecting relevant studies for inclusion in the review of quality indicator development in the intensive care unit. The initial search identified 670 records. After screening and a full-text eligibility assessment, 14 studies were included in the final review

We identified 670 articles in the initial search, and after screening abstracts, selected 69 studies for full text review. We included 14 articles as sources of QIs [2, 8, 10–21]. The study flow diagram is shown in Fig. 1. We also reviewed the evidence-based guidelines published by the Japanese Society of Intensive Care Medicine [22, 23] and leading international guidelines [24–28]. After a screening process, 44 potential QIs were extracted for evaluation. Table 1 shows the details of extracted potential QIs.

**Table 1:** Description of the potential quality indicators prepared for the first-round evaluation

No	Quality indicator	Denominator to calculate the QI (criteria for applicable patients)	Numerator to calculate the QI (criteria for achievement)	Type of QI
1	ICU mortality	Patients discharged from the ICU	Patients who die during the ICU stay	Outcome
2	Hospital mortality	Patients who are admitted to the ICU during their hospitalization and are subsequently discharged from the hospital	Patients who die during the hospital stay	Outcome
3	30-day mortality	Patients discharged from the ICU	Patients who died within 30 days of ICU admission	Outcome
4	Standardized mortality rate	Mean predicted mortality rate	Observed mortality rate	Outcome
5	Readmission to the ICU	Patients discharged alive from the ICU	Patients with an unplanned ICU readmission within 72 h of discharge	Outcome
6	Nighttime ICU discharge	Patients discharged alive from the ICU	Patients discharged from the ICU between 22:00 and 06:59	Outcome
7	ICU bed occupancy rate	Number of ICU beds × 365 days	Number of patients admitted to the ICU in 1 year	Structure
8	ICU stay lasting 7 days or more	Patients discharged from the ICU	Patients who stayed in the ICU for 7 days or more	Outcome
9	Length of stay in ICU	Patients discharged from the ICU	Sum of the length of stay for all patients discharged from the ICU	Outcome
10	Full ICU occupation rate	Total number of days in a year (365)	Number of days in a year the ICU was fully occupied	Outcome
11	Targeted temperature management after cardiac arrest	Patients discharged from the ICU after cardiopulmonary resuscitation	Patients who received therapeutic hypothermia	Outcome
12	Monitoring sedation	N/A	The facility has an established policy and procedure for sedation monitoring	Structure
13	Monitoring pain	N/A	The facility has an established policy and procedure for pain monitoring	Structure
14	Identification of delirium	N/A	The facility has an established policy and procedure for delirium monitoring	Structure
15	Reintubation	Mechanically ventilated patients discharged from the ICU (excluding those with a tracheostomy)	Patients who were reintubated within 48 h of extubation	Outcome
16	Duration of mechanical ventilation	Mechanically ventilated patients discharged from the ICU	Sum of the duration of mechanical ventilation for all ventilated patients	Outcome
17	Prolonged mechanical ventilation	Mechanically ventilated patients discharged from the ICU	Patients who received mechanical ventilation for 10 days or more	Outcome
18	Postoperative respiratory failure	Postoperative patients admitted to and discharged from the ICU	Patients who received mechanical ventilation for 10 days or more after surgery	Outcome
19	Unplanned extubation	Total number of mechanical ventilation days for all ventilated patients	Patients who had an unplanned extubation	Outcome
20	Use of a weaning protocol	N/A	The facility has an established policy and procedure for weaning from mechanical ventilation	Structure
21	Ventilator-associated pneumonia (VAP) prevention bundle	Mechanically ventilated patients discharged from the ICU	Patients for whom the VAP prevention bundle was adhered to	Process
22	Low tidal volume ventilation in ARDS	Patients with ARDS who received mechanical ventilation in the ICU and were discharged from the ICU	Patients managed with a tidal volume of 6–8 mL/kg (predicted body weight) and an inspiratory plateau pressure < 30 cmH2O	Process
23	Early enteral nutrition	Patients who stayed in the ICU for 48 h or more and were subsequently discharged	Patients who received enteral nutrition within 48 h of ICU admission	Process
24	Stress ulcer prophylaxis	Patients discharged from the ICU	Patients who received a proton pump inhibitor (PPI) or H2 blocker	Process
25	Stress ulcer prophylaxis during mechanical ventilation	Mechanically ventilated patients discharged from the ICU	Patients who received a proton pump inhibitor (PPI) or H2 blocker	Process
26	Glycemic control	Total number of blood glucose measurements for all patients during their ICU stay	Number of measurements, where blood glucose was ≤ 80 mg/dL or ≥ 200 mg/dL	Process
27	Blood culture sampling	Non-postoperative patients who received antibiotics and were discharged from the ICU	Patients who had blood cultures drawn before the initiation of antibiotics	Process
28	Prevalence of MRSA	Patients discharged from the ICU	Patients with MRSA detected from a specimen collected within 24 h of ICU admission	Outcome
29	New-onset MRSA	Patients discharged from the ICU	Patients with new-onset MRSA detected more than 24 h after ICU admission	Outcome
30	New-onset multidrug-resistant organisms	Patients discharged from the ICU	Patients with a new multidrug-resistant organism (resistant to ≥ 2 agents) detected more than 24 h after ICU admission	Outcome
31	Restrictive transfusion strategy in sepsis	Patients with sepsis discharged from the ICU (excluding those with myocardial infarction, severe hypoxemia, or acute hemorrhage)	Patients who received RBC transfusion only when their hemoglobin level was below 7 g/dL	Process
32	Red blood cell (RBC) transfusion administration	Patients discharged from the ICU	Patients who received an RBC transfusion	Process
33	Amount of RBC transfusion	Patients who received an RBC transfusion and were discharged from the ICU	Total units of RBCs transfused	Process
34	Plasma transfusion administration	Patients discharged from the ICU	Patients who received a plasma transfusion	Process
35	Amount of plasma transfusion	Patients who received a plasma transfusion and were discharged from the ICU	Total units of plasma transfused	Process
36	Venous thromboembolism (VTE) prophylaxis	Patients discharged from the ICU	Patients who received anticoagulation therapy for VTE prophylaxis	Process
37	Hand hygiene	Number of observed hand hygiene opportunities during audit observations (e.g., WHO 5 Moments for Hand Hygiene)	Number of observed opportunities in which hand hygiene was performed appropriately	Process
38	Early rehabilitation	Patients discharged from the ICU	Patients who started rehabilitation within 48 h of ICU admission	Process
39	Dedicated intensivist hours	N/A	The number of hours an intensivist is dedicated to the ICU	Structure
40	Patient-to-nurse ratio	N/A	The number of patients assigned to one nurse	Structure
41	Multidisciplinary rounds and daily goal setting	N/A	Multidisciplinary rounds and daily goal setting are implemented	Structure
42	Standardized handoff communication	N/A	A standardized handoff method is in place	Structure
43	Adverse event reporting system	N/A	A system for reporting adverse events is in place	Structure
44	Incident reporting by physicians	Total number of incident reports for ICU patients	Number of incident reports submitted by physicians for ICU patients	Structure

N/A: Not applicable; ICU: Intensive Care Unit; QI: Quality Indicator; VAP: Ventilator-associated pneumonia; ARDS: Acute Respiratory Distress Syndrome; MRSA: Methicillin-Resistant Staphylococcus Aureus; RBC: Red Blood Cell; VTE: Venous Thromboembolism; WHO: World Health Organization

### First questionnaire round

In the first evaluation round, conducted between 1st May and 31st May 2018, the fourteen expert panel members rated the 44 potential QIs. Of the 44 initial candidates, the 34 indicators had a median appropriateness score of 7 or higher. Among these 34 indicators, 33 showed agreement and were classified as “Appropriate”, while the one remaining indicator had disagreement and, therefore, classified as “Uncertain”. Ten indicators had median scores between 5 and 6.5, and were all classified as “Uncertain”. No indicators were classified as “Inappropriate”. The 11 indicators rated as “Uncertain” were prioritized for discussion at the face-to-face panel meeting (Table 2).

**Table 2:** Appropriateness ratings and final decisions for each quality indicator

No	Quality indicator	Round 1		Meeting decision	Round 2		Final decision
		Median rating	Agreement/disagreement		Median rating	Agreement/disagreement	
1	ICU mortality	9	Agreement	Appropriate	9	Agreement	Selected
2	Hospital mortality	7.5	Agreement	Appropriate	9	Agreement	Selected
3	30-day mortality	7	Agreement	Appropriate	7.5	Agreement	Selected
4	Standardized mortality rate	8	Agreement	Appropriate	9	Agreement	Selected
5	Readmission to the ICU	8	Agreement	Appropriate	8	Agreement	Selected
6	Nighttime ICU discharge	6	Agreement	Deleted	–	–	–
7	ICU bed occupancy rate	6	Agreement	Uncertain	7	Agreement	Selected
8	ICU stay lasting 7 days or more	7	Agreement	Appropriate	7	Agreement	Selected
9	Length of stay in ICU	7	Agreement	Appropriate	7	Agreement	Selected
10	Full ICU occupation rate	5	Agreement	Deleted	–	–	–
11	Targeted temperature management after cardiac arrest	7	Disagreement	Uncertain	6	Agreement	Deleted
12	Monitoring sedation	9	Agreement	Appropriate	9	Agreement	Selected
13	Monitoring pain	9	Agreement	Appropriate	9	Agreement	Selected
14	Identification of delirium	9	Agreement	Appropriate	9	Agreement	Selected
15	Reintubation	7.5	Agreement	Appropriate	7	Agreement	Selected
16	Duration of mechanical ventilation	8	Agreement	Appropriate	8	Agreement	Selected
17	Prolonged mechanical ventilation	6.5	Agreement	Uncertain	7.5	Agreement	Selected
18	Postoperative respiratory failure	7	Agreement	Modified	7.5	Agreement	Selected
19	Unplanned extubation	8	Agreement	Appropriate	7	Agreement	Selected
20	Use of a weaning protocol	7.5	Agreement	Appropriate	8	Agreement	Selected
21	Ventilator-associated pneumonia (VAP) prevention bundle	8	Agreement	Appropriate	8	Agreement	Selected
22	Low tidal volume ventilation in ARDS	8	Agreement	Appropriate	7.5	Agreement	Selected
23	Early enteral nutrition	7	Agreement	Appropriate	7.5	Agreement	Selected
24	Stress ulcer prophylaxis	6.5	Agreement	Uncertain	6	Agreement	Deleted
25	Stress ulcer prophylaxis during mechanical ventilation	8	Agreement	Modified	7	Agreement	Selected
26	Glycemic control	7	Agreement	Appropriate	7	Agreement	Selected
27	Blood culture sampling	9	Agreement	Appropriate	8	Agreement	Selected
28	Prevalence of MRSA	6	Agreement	Deleted	–	–	–
29	New-onset MRSA	8	Agreement	Appropriate	7	Agreement	Selected
30	New-onset multidrug-resistant organisms	7	Agreement	Appropriate	7	Agreement	Selected
31	Restrictive transfusion strategy in sepsis	7	Agreement	Appropriate	6.5	Agreement	Selected
32	Red blood cell (RBC) transfusion administration	6	Agreement	Deleted	–	–	–
33	Amount of RBC transfusion	5	Agreement	Deleted	–	–	–
34	Plasma transfusion administration	5	Agreement	Deleted	–	–	–
35	Amount of plasma transfusion	5	Agreement	Deleted	–	–	–
36	Venous thromboembolism (VTE) prophylaxis	7	Agreement	Modified	8	Agreement	Selected
37	Hand hygiene	8.5	Agreement	Appropriate	8	Agreement	Selected
38	Early rehabilitation	8	Agreement	Appropriate	8	Agreement	Selected
39	Dedicated intensivist hours	8	Agreement	Appropriate	8	Agreement	Selected
40	Patient-to-nurse ratio	8	Agreement	Appropriate	8	Agreement	Selected
41	Multidisciplinary rounds and daily goal setting	8	Agreement	Appropriate	8	Agreement	Selected
42	Standardized handoff communication	7	Agreement	Appropriate	7.5	Agreement	Selected
43	Adverse event reporting system	7	Agreement	Appropriate	7.5	Agreement	Selected
44	Incident reporting by physicians	7	Agreement	Appropriate	7.5	Agreement	Selected
45	Ventilator-associated pneumonia (VAP)	–	–	Added	8	Agreement	Selected
46	Number of clinical engineers	–	–	Added	7	Agreement	Selected
47	Number of pharmacists	–	–	Added	7	Agreement	Selected

ICU: Intensive Care Unit; VAP: Ventilator-associated pneumonia; ARDS: Acute Respiratory Distress Syndrome; MRSA: Methicillin-Resistant Staphylococcus Aureus; RBC: Red Blood Cell; VTE: Venous Thromboembolism

### Expert panel meeting

The face-to-face panel meeting was held on 13th June 2018. During the meeting, the results of the first questionnaire round were presented, and all QIs were discussed based on the predetermined criteria, such as clinical importance, scientific evidence, and feasibility of measurement in the ICU setting. As a result of the discussion, 3 of the 44 potential indicators were modified, 7 were deleted, and 3 new indicators were added by the panel members. Reasons for modification or deletion are summarized in Table 3 [29, 30]. The primary reasons for deletion included a lack of clear evidence, difficulty in data collection from electronic health records, and overlap with other indicators.

**Table 3:** Reasons for modifications and deletions of quality indicators after the expert panel meeting

No	Quality indicator	Status	Reasons for modification or deletion
1	Nighttime ICU discharge	Deleted	The panel concluded that nighttime discharge does not necessarily reflect poor quality of care and can be clinically appropriate in certain situations
2	Full ICU occupation rate	Deleted	This indicator was considered to be more related to hospital management and resource allocation than to the direct quality of clinical care
3	Targeted temperature management after cardiac arrest	Deleted	Deleted, because recent clinical evidence [e.g., the TTM trial [29, 30]] suggests that targeted normothermia is an acceptable alternative to hypothermia. Therefore, the panel concluded that mandating hypothermia alone as a quality indicator is not aligned with current evidence-based practice
4	Postoperative respiratory failure	Modified	The definition was modified to improve specificity and fairness. The denominator was narrowed to include only patients after elective surgery. In addition, the time threshold for defining respiratory failure was changed from 10 days to 96 h, as the panel concluded the 10-day period lacked a strong evidence base
5	Stress ulcer prophylaxis	Deleted	Deleted, because the panel concluded there is a lack of evidence for routine prophylaxis in all ICU patients. It was agreed that a more appropriate indicator would target high-risk patients for whom prophylaxis is recommended, such as those on mechanical ventilation
6	Stress ulcer prophylaxis during mechanical ventilation	Modified	The definition was refined as the denominator was narrowed to target a more specific high-risk population by including only patients on mechanical ventilation for 48 h or more
7	Prevalence of MRSA	Deleted	This indicator was considered to reflect community-acquired infections or issues at a previous hospital rather than the quality of care within the ICU itself
8	Red blood cell (RBC) transfusion administration	Deleted	Deleted, because the panel concluded that the act of administering an RBC transfusion itself does not reflect the quality of care, and there is no evidence to support its use as a quality indicator
9	Amount of RBC transfusion	Deleted	The panel decided that the more critical process indicator is the transfusion strategy, not the total volume of blood transfused
10	Plasma transfusion administration	Deleted	Deleted, because the panel concluded that the act of administering a plasma transfusion itself does not reflect the quality of care, and there is no evidence to support its use as a quality indicator
11	Amount of plasma transfusion	Deleted	Similar to RBCs, the appropriateness of the transfusion decision was deemed more important than the total volume, and evidence for a specific volume target is lacking
12	Venous thromboembolism (VTE) prophylaxis	Modified	The indicator was broadened to include mechanical prophylaxis (e.g., intermittent pneumatic compression and elastic stockings) in addition to pharmacological therapy
13	Ventilator-associated pneumonia (VAP) per 1,000 ventilator-days	Added	Added, because VAP is a clinically important ICU-acquired complication and a widely used outcome metric for quality benchmarking; it complements the existing process indicator (VAP prevention bundle) and can be operationalized using an established surveillance definition (e.g., JANIS)
14	Number of clinical engineers	Added	Added to reflect Japan-specific ICU staffing and safety systems, where clinical engineers play a key role in supporting safe operation and management of life-support devices; this structural element is measurable via an institutional (facility-level) survey
15	Number of pharmacists	Added	Added, because ICU-dedicated pharmacists contribute to medication safety and quality improvement activities (e.g., review/optimization of pharmacotherapy); this structural element is measurable via an institutional (facility-level) survey

The modification/deletion decisions summarized in Table 3 were made during the 2018 face-to-face panel meeting based on the evidence available at that time. Where later publications are cited in the rationale [e.g., the TTM2 trial published in 2021 [30]], these references were provided to contextualize how the evidence base subsequently evolved and/or to reflect information considered during the 2025 re-evaluation; such evidence was not available to the panel at the time of the 2018 meeting

ICU: Intensive Care Unit; MRSA: Methicillin-Resistant Staphylococcus Aureus; RBC: Red Blood Cell; VAP: Ventilator-associated pneumonia

### Second questionnaire round and finalization of the 2018 consensus set (round 2)

The revised list of 40 QIs (including modified, retained, and newly added indicators) was sent to the panel members for the second and final rating round. In this round, 37 indicators had a median score of 7 or higher, with the remaining three scoring between 6 and 6.5. All indicators reached panel agreement. Consequently, 37 indicators were deemed “Appropriate” and three “Uncertain”. After the final evaluation, two indicators were deleted (Table 2). As a sensitivity analysis, disagreement was also assessed using the IPRAS method; the appropriateness classification did not change compared with the primary count-based definition (Supplementary Table S1).

Ultimately, a final set of 38 QIs for quality of care in the ICU was established. Of these, 13 are structure indicators, 10 are process indicators, and 15 are outcome indicators. Most selected QIs can be measured using data available from existing electronic health records and administrative claims data, while a subset requires additional data sources, such as facility-level surveys or direct observation (Table 4). The 2018 consensus set of selected QIs is shown in Table 4 [31, 32].

**Table 4:** Final set of selected quality indicators for ICU care

No	Quality indicator	Denominator to calculate the QI (criteria for applicable patients)	Numerator to calculate the QI (criteria for achievement)	Type of QI	Data sources
1	ICU mortality	Patients discharged from the ICU	Patients who die during the ICU stay	Outcome	JIPAD, Administrative claims data
2	Hospital mortality	Patients who are admitted to the ICU during their hospitalization and are subsequently discharged from the hospital	Patients who die during the hospital stay	Outcome	JIPAD, Administrative claims data
3	30-day mortality	Patients discharged from the ICU	Patients who died within 30 days of ICU admission	Outcome	JIPAD, Administrative claims data
4	Standardized mortality rate	Mean predicted mortality rate calculated by severity scores, such as APACHE II	Observed mortality rate	Outcome	JIPAD
5	Readmission to the ICU	Patients discharged alive from the ICU	Patients with an unplanned ICU readmission within 72 h of discharge	Outcome	JIPAD, Administrative claims data
6	ICU bed occupancy rate	Number of staffed ICU beds × 365 days	Total number of ICU patient-days during the year	Structure	Administrative claims data
7	ICU stay lasting 7 days or more	Patients discharged from the ICU	Patients who stayed in the ICU for 7 days or more	Outcome	JIPAD, Administrative claims data
8	Length of stay in ICU	Patients discharged from the ICU	Sum of the length of stay for all patients discharged from the ICU	Outcome	JIPAD, Administrative claims data
9	Monitoring sedation	N/A	The facility has an established policy and procedure for sedation monitoring	Structure	Survey data
10	Monitoring pain	N/A	The facility has an established policy and procedure for pain monitoring	Structure	Survey data
11	Identification of delirium	N/A	The facility has an established policy and procedure for delirium monitoring	Structure	Survey data
12	Reintubation	Mechanically ventilated patients discharged from the ICU (excluding those with a tracheostomy)	Patients who were reintubated within 48 h of extubation	Outcome	JIPAD, Administrative claims data
13	Duration of mechanical ventilation	Mechanically ventilated patients discharged from the ICU	Sum of the duration of mechanical ventilation for all ventilated patients	Outcome	JIPAD, Administrative claims data
14	Prolonged mechanical ventilation	Mechanically ventilated patients discharged from the ICU	Patients who received mechanical ventilation for 10 days or more	Outcome	JIPAD, Administrative claims data
15	Postoperative respiratory failure	Postoperative patients admitted to and discharged from the ICU after elective surgery	Patients who received mechanical ventilation for 96 h or more after surgery	Outcome	JIPAD
16	Unplanned extubation	Total number of mechanical ventilation-days for all ventilated patients	Patients who had an unplanned extubation	Outcome	Electronic health records
17	Use of a weaning protocol	N/A	The facility has an established policy and procedure for weaning from mechanical ventilation	Structure	Survey data
18	Ventilator-associated pneumonia (VAP) prevention bundle	Patients who received mechanical ventilation and were discharged from the ICU	Patients for whom the VAP prevention bundle was adhered to	Process	Electronic health records
19	Ventilator-associated pneumonia (VAP) per 1,000 ventilator-days	Total number of ventilator-days in the ICU	Number of VAP events (according to the JANIS definition [31, 32]* VAP rate (per 1,000 ventilator-days): (Numerator/Denominator) × 1000	Outcome	Electronic health records
20	Low tidal volume ventilation in ARDS	Patients with ARDS who received mechanical ventilation in the ICU and were discharged from the ICU	Patients managed with a tidal volume of 6–8 mL/kg (predicted body weight) and an inspiratory plateau pressure < 30 cmH2O	Process	Electronic health records
21	Early enteral nutrition	Patients who stayed in the ICU for 48 h or more and were subsequently discharged	Patients who received enteral nutrition within 48 h of ICU admission	Process	Administrative claims data
22	Stress ulcer prophylaxis during mechanical ventilation	Mechanically ventilated patients for 48 h or more who were discharged from the ICU	Patients who received a proton pump inhibitor (PPI) or H2 blocker	Process	Administrative claims data
23	Glycemic control	Total number of blood glucose measurements for all patients during their ICU stay	Number of measurements, where blood glucose was ≤ 80 mg/dL or ≥ 200 mg/dL	Process	Laboratory data
24	Blood culture sampling	Non-postoperative patients who received antibiotics and were discharged from the ICU	Patients who had blood cultures drawn before the initiation of antibiotics	Process	Administrative claims data
25	New-onset MRSA	Patients discharged from the ICU	Patients with new-onset MRSA detected more than 24 h after ICU admission	Outcome	Electronic health records
26	New-onset multidrug-resistant organisms	Patients discharged from the ICU	Patients with a new multidrug-resistant organism (resistant to ≥ 2 agents) detected more than 24 h after ICU admission	Outcome	Electronic health records
27	Restrictive transfusion strategy in sepsis	Patients with sepsis discharged from the ICU (excluding those with myocardial infarction, severe hypoxemia, or acute hemorrhage)	Patients who received RBC transfusion only when their hemoglobin level was below 7 g/dL	Process	Electronic health records
28	Venous thromboembolism (VTE) prophylaxis	Patients discharged from the ICU	Patients who received pharmacological or mechanical anticoagulation therapy for VTE prophylaxis	Process	Administrative claims data
29	Hand hygiene	Number of observed hand hygiene opportunities during audit observations (e.g., WHO 5 Moments for Hand Hygiene)	Number of observed opportunities in which hand hygiene was performed appropriately	Process	Facility-reported results from locally audited monitoring
30	Early rehabilitation	Patients discharged from the ICU	Patients who started rehabilitation within 48 h of ICU admission	Process	Administrative claims data
31	Dedicated intensivist hours	N/A	The number of hours an intensivist is dedicated to the ICU	Structure	Survey data
32	Patient-to-nurse ratio	N/A	The number of patients assigned to one nurse	Structure	Survey data
33	Multidisciplinary rounds and daily goal setting	N/A	Multidisciplinary rounds and daily goal setting are implemented at least 5 days per week	Structure	Survey data
34	Standardized handoff communication	N/A	A standardized handoff method is in place for all ICU shift changes and inter-unit transfers	Structure	Survey data
35	Adverse event reporting system	N/A	A system for reporting adverse events is in place	Structure	Survey data
36	Incident reporting by physicians	Total number of incident reports for ICU patients	Number of incident reports submitted by physicians for ICU patients	Structure	Survey data
37	Number of clinical engineers	N/A	Number of clinical engineers dedicated to the ICU during weekday day shifts	Structure	Survey data
38	Number of pharmacists	N/A	Number of pharmacists dedicated to the ICU during weekday day shifts	Structure	Survey data

In this study, “Survey data” refer to institutional (facility-level) surveys (e.g., staffing, protocols, and safety systems) rather than patient-reported surveys

ICU: Intensive Care Unit, QI: Quality Indicator, JIPAD: the Japanese Intensive care PAtient Database, N/A: Not applicable, VAP: Ventilator-associated pneumonia, JANIS: Japan Nosocomial Infection Surveillance, ARDS: Acute Respiratory Distress Syndrome, MRSA: Methicillin-Resistant Staphylococcus Aureus, RBC: Red Blood Cell, VTE: Venous Thromboembolism

### Re-evaluation round (round 3; 2025 update)

In 2025, all 14 original panelists participated in Round 3 and returned complete ratings (14/14, 100%). The expert panel re-rated all indicators from the 2018 consensus set using the same nine-point scale and classification rules. Of the original 38 indicators, 37 remained classified as “Appropriate”, whereas one indicator (restrictive transfusion strategy in sepsis) was reclassified as “Uncertain” (median 6) and was, therefore, not retained in the updated core set.

In addition, one newly proposed indicator reflecting updated international practice (No. 39, ventilator-associated events per 1,000 ventilator-days) was proposed for rating, rated as “Appropriate” (median 8) and was included in the updated set. No indicator met the criterion for disagreement using either the primary count-based definition or the IPRAS definition; the appropriateness classification was unchanged when using the IPRAS-based definition (Supplementary Table S2). Round 3 re-evaluation results and final status are shown in Table 5 [33, 34]. Changes in median appropriateness scores and rating distributions between Round 2 (2018) and Round 3 (2025) are summarized in Supplementary Table S4.

**Table 5:** Updated final quality indicators status after the 2025 re-evaluation (Round 3)

No	Quality indicator	Type of QI	Round 3		Final status
			Median rating	Appropriateness classification	
1	ICU mortality	Outcome	9	Appropriate	Retained
2	Hospital mortality	Outcome	9	Appropriate	Retained
3	30-day mortality	Outcome	8	Appropriate	Retained
4	Standardized mortality rate	Outcome	9	Appropriate	Retained
5	Readmission to the ICU	Outcome	8	Appropriate	Retained
6	ICU bed occupancy rate	Structure	7	Appropriate	Retained
7	ICU stay lasting 7 days or more	Outcome	7	Appropriate	Retained
8	Length of stay in ICU	Outcome	8	Appropriate	Retained
9	Monitoring sedation	Structure	9	Appropriate	Retained
10	Monitoring pain	Structure	9	Appropriate	Retained
11	Identification of delirium	Structure	9	Appropriate	Retained
12	Reintubation	Outcome	7	Appropriate	Retained
13	Duration of mechanical ventilation	Outcome	8	Appropriate	Retained
14	Prolonged mechanical ventilation	Outcome	8	Appropriate	Retained
15	Postoperative respiratory failure	Outcome	7.5	Appropriate	Retained
16	Unplanned extubation	Outcome	7.5	Appropriate	Retained
17	Use of a weaning protocol	Structure	8.5	Appropriate	Retained
18	Ventilator-associated pneumonia (VAP) prevention bundle	Process	8	Appropriate	Retained
19	Ventilator-associated pneumonia (VAP) per 1,000 ventilator-days	Outcome	8	Appropriate	Retained
20	Low tidal volume ventilation in ARDS	Process	8	Appropriate	Retained
21	Early enteral nutrition	Process	8	Appropriate	Retained
22	Stress ulcer prophylaxis during mechanical ventilation	Process	8	Appropriate	Retained
23	Glycemic control	Process	8	Appropriate	Retained
24	Blood culture sampling	Process	8	Appropriate	Retained
25	New-onset MRSA	Outcome	7	Appropriate	Retained
26	New-onset multidrug-resistant organisms	Outcome	7.5	Appropriate	Retained
27	Restrictive transfusion strategy in sepsis	Process	6	Uncertain	Not Retained
28	Venous thromboembolism (VTE) prophylaxis	Process	8	Appropriate	Retained
29	Hand hygiene	Process	8	Appropriate	Retained
30	Early rehabilitation	Process	8	Appropriate	Retained
31	Dedicated intensivist hours	Structure	8	Appropriate	Retained
32	Patient-to-nurse ratio	Structure	8	Appropriate	Retained
33	Multidisciplinary rounds and daily goal setting	Structure	8	Appropriate	Retained
34	Standardized handoff communication	Structure	8	Appropriate	Retained
35	Adverse event reporting system	Structure	8	Appropriate	Retained
36	Incident reporting by physicians	Structure	8	Appropriate	Retained
37	Number of clinical engineers	Structure	8	Appropriate	Retained
38	Number of pharmacists	Structure	8	Appropriate	Retained
39	Ventilator-associated events (VAE) per 1,000 ventilator days	Outcome	8	Appropriate	New

1. Operational definitions for retained indicators are provided in Table 4

2. No. 27 was reclassified as Uncertain in Round 3 (median 6) and was, therefore, not retained in the updated core set

3. No. 39 (VAE) was newly proposed in Round 3 to reflect updated international practice, where VAE surveillance is increasingly used alongside VAP

4. The operational definition for the newly proposed indicator (No. 39 VAE) is as follows [33, 34]; Denominator: Number of ventilator-days in the same location during the surveillance period. Numerator: Number of ventilator-associated events (VAEs) identified in the surveillance location during the surveillance period. VAE rate (per 1,000 ventilator-days): (Numerator / Denominator) × 1,000

5. No indicators met the criterion for disagreement using either the count-based definition or the IPRAS definition (Supplementary Method 2)

6. This table also reports the indicator reclassified as Uncertain for transparency

ICU: Intensive Care Unit; VAP: Ventilator-associated pneumonia; ARDS: Acute Respiratory Distress Syndrome; MRSA: Methicillin-Resistant Staphylococcus Aureus; VTE: Venous Thromboembolism; VAE: Ventilator-associated events

## Discussion

In this study, we developed a comprehensive set of quality indicators for adult intensive care in Japan using a rigorous, evidence-based modified RAND/UCLA appropriateness method, and further updated this set through a formal re-evaluation round conducted in 2025. The original consensus process in 2017–2018 yielded 38 indicators, which were well-balanced across Donabedian’s domains (13 structure, 10 process and 15 outcome) and were designed with a strong emphasis on measurability, prioritizing indicators derivable from existing electronic health records and administrative claims data while also incorporating key indicators that require other data sources, such as facility-level surveys or direct observation. In the 2025 re-evaluation, the expert panel confirmed the continued appropriateness of most indicators, did not retain one indicator as a core QI, and added a contemporary surveillance indicator (VAE per 1,000 ventilator-days), resulting in an updated 2025 core set of 38 indicators. To our knowledge, this is the first study to develop a comprehensive, evidence-based set of indicators for clinical quality in Japanese ICUs. Together, these findings represent a crucial step toward standardizing quality assessment and promoting continuous quality improvement in Japanese ICUs.

The only indicator not retained as a core QI in the 2025 re-evaluation was restrictive transfusion strategy in sepsis, because transfusion thresholds in sepsis are often individualized according to patient characteristics, hemodynamics, and bleeding/ischemia risk, making a single-threshold indicator prone to exceptions and potential misclassification. Nonetheless, transfusion practice remains an important domain and warrants reconsideration in future updates.

The development of QIs for the care of patients in ICUs has been a global endeavor for decades, with multiple organizations and national initiatives proposing domains and indicators for quality measurement in critical care [2, 14, 28, 35–40]. This study aligns with these efforts by applying a structured consensus methodology and addressing fundamental aspects of ICU quality, including outcomes (e.g., mortality), safety, and prevention of healthcare-associated complications. Importantly, we extended the original 2017–2018 consensus by conducting a re-evaluation round in 2025 (Round 3), which confirmed the stability of the core set while allowing targeted refinement: one indicator was not retained as a core QI, and a contemporary surveillance indicator (ventilator-associated events, VAE) was incorporated.

To facilitate interpretation by an international audience and to enable a systematic comparison with existing international frameworks, we created Supplementary Table S3 as an international mapping table. The table maps each Japanese ICU QI to major international sources and indicates whether the correspondence is an exact match, a conceptual match within the same domain, or domain-level alignment (e.g., WHO patient safety/IPC/AMR). Overall, the comparison shows substantial alignment with global priorities while also clarifying indicators that reflect the Japanese context, especially those related to staffing and safety systems.

Even when the overarching quality domains are shared internationally, the feasibility of measurement and implementation depends heavily on local data infrastructure and health-system design. In Japan, ICU quality measurement can leverage the national ICU registry (JIPAD) and administrative claims data (DPC), but the availability, structure, and linkage of these data differ from those in other countries. In addition, the practical uptake of QIs is influenced by domestic governance structures, including facility requirements and reimbursement-related documentation, which makes explicit data-source specification and feasibility assessment particularly important in Japan. Accordingly, our indicator definitions and data-source specifications emphasize pragmatic measurability in the Japanese setting, including a transparent distinction between indicators measurable from routinely available electronic data and those requiring facility-level surveys. In addition, several indicators reflect the Japanese ICU staffing and regulatory environment (e.g., specific professional roles and institutional safety structures), which may not be specified as formal indicators in international “core sets” but are relevant to quality and safety within Japan. For certain indicators (e.g., glycemic control), we chose operational definitions that can be measured consistently across institutions using routinely available data; more granular metrics such as time-in-range may be desirable but are not always feasible in real-world data sets with heterogeneous measurement frequency.

Some context-specific indicators were proposed during the face-to-face panel meeting rather than being included in the initial candidate list before the first rating round. In our process, the initial list was anchored in literature-derived indicators and guideline-based recommendations to ensure a comprehensive evidence-based baseline. The subsequent multidisciplinary discussion made local feasibility and practice structures more explicit and enabled the panel to propose additional indicators reflecting the Japanese ICU environment. Nevertheless, future updates could strengthen contextualization earlier in the process by incorporating a structured “context scan” before the first rating round (e.g., systematic mapping to domestic regulatory/reimbursement requirements and stakeholder input), so that context-specific candidate indicators are considered from the outset.

A key strength of this study is the rigorous and reproducible consensus methodology based on the modified RAND/UCLA appropriateness method, supported by a multidisciplinary panel with broad geographic and institutional representation. We also prioritized feasibility by providing clear operational definitions and specifying realistic data sources for each indicator, distinguishing routinely available electronic data from facility-level surveys and other sources. In addition, our sensitivity analysis using the IPRAS-based disagreement definition yielded the same overall classification as the predefined count-based definition, supporting the robustness of the consensus classification approach.

The value of these QIs will ultimately depend on implementation and ongoing evaluation in real-world practice. A national measurement framework leveraging JIPAD and administrative claims data could enable routine benchmarking and feedback to participating ICUs, supporting continuous quality improvement. Future work should also evaluate the construct and predictive validity of this QI set by assessing whether higher achievement is associated with improved patient outcomes and safety, and by examining measurement reliability across institutions. As an example of how QI performance can be linked to outcomes, prior literature (e.g., Wu et al.) has reported associations between quality measurement and patient outcomes in other settings [41, 42]; however, ICU-specific validation of our indicator set remains an important next step. These efforts are essential to translate a consensus-based indicator set into measurable improvements in ICU care. Some indicators require careful interpretation as quality signals. For example, reintubation rates should be interpreted alongside balancing measures (e.g., ventilator-free days or sedation/weaning practices), because an extremely low reintubation rate is not necessarily optimal if it reflects delayed extubation or overly conservative practice.

Several limitations warrant consideration. First, although the original consensus development was conducted in 2017–2018, we addressed the substantial time gap by performing a formal re-evaluation in 2025 using the same rating and classification framework. This approach confirmed the robustness of the core set while allowing targeted updating; however, continued periodic review will remain necessary as evidence and practice evolve.

Second, the panel consisted of Japanese healthcare professionals, which was appropriate for contextual tailoring but may limit generalizability to other health systems. Third, our process did not include patients or family representatives. Patient- and family-centered outcomes, including patient-reported outcome/experience measures and longer term recovery after critical illness (e.g., post-intensive care syndrome-related domains), are increasingly emphasized internationally [43, 44]. These emerging domains were not explicitly included in the current core set and should be prioritized in future updates, potentially through inclusion of patient representatives and complementary qualitative work. Fourth, for indicators relying on facility-reported audit results (e.g., hand hygiene), audit implementation details (sampling, frequency, and assessor training) were not standardized in the original consensus process and may vary across institutions; thus, measurement and reporting bias should be considered when using these metrics for benchmarking.

Despite these limitations, this study provides a feasible and contextually adapted ICU QI set for Japan and an essential foundation for national benchmarking and quality improvement. The updated core set, together with clear operational definitions and specified data sources, can support future implementation using routinely available data and facilitate iterative refinement as evidence and practice evolve.

## Conclusion

In conclusion, we developed a feasible, contextually adapted ICU QI set for Japan using a rigorous RAND/UCLA-based consensus process and confirmed its current validity through a 2025 re-evaluation round. By providing an updated core set (Table 5) and an explicit international crosswalk (Supplementary Table S3), this work offers a practical foundation for national benchmarking and continuous quality improvement while enabling transparent comparison with international standards. This framework also establishes a platform for iterative refinement as clinical evidence, data infrastructure, and societal expectations continue to evolve.

## Supplementary Information

Supplementary Material 1.

## Data Availability

No datasets were generated or analyzed during the current study.

## Abbreviations

ICU: Intensive care unit
QI: Quality indicator
ESICM: European Society of Intensive Care Medicine
JIPAD: The Japanese Intensive care PAtient Database
IPRAS: Interpercentile range adjusted for symmetry
VAP: Ventilator-associated pneumonia
VAE: Ventilator-associated events
ARDS: Acute Respiratory Distress Syndrome
MRSA: Methicillin-Resistant Staphylococcus Aureus
RBC: Red Blood Cell
VTE: Venous Thromboembolism
WHO: World Health Organization
DPC: The Diagnosis Procedure Combination
