# Supplementary material for: Development and 2025 re-evaluation of a Japanese quality indicator set for adult intensive care: a modified RAND/UCLA Delphi study
Source: J Intensive Care. 2026 Feb 10;14:29. doi: 10.1186/s40560-026-00863-w (PMC12990454; doi:10.1186/s40560-026-00863-w)
Supplement: Supplementary file 1 — Supplementary Material 1. [file 40560_2026_863_MOESM1_ESM.docx]

**Supplement Methods 1. Search strategy**

Full search strategies for MEDLINE (via PubMed)

#1 quality indicators, health care [MeSH Terms]

#2 “clinical indicator” OR “clinical indicators”

#3 “process indicator” OR “process indicators”

#4 “performance indicator” OR “performance indicators”

#5 #1 OR #2 OR #3 OR #4

#6 critical care [MeSH Terms]

#7 intensive care unit [MeSH Terms]

#8 #6 OR #7

#9 #5 AND #8

Searched in 21:12 November 6, 2017.

670 articles

**Supplementary Methods 2. Interpercentile Range Adjusted for Symmetry (IPRAS) sensitivity analysis**

As a sensitivity analysis, we assessed disagreement using the interpercentile range adjusted for symmetry (IPRAS) method described in the RAND/UCLA Appropriateness Method User’s Manual. For each indicator, 14 panelists provided ratings on a 9-point Likert scale. We first calculated the 30th percentile (P30) and 70th percentile (P70) of the 14 ratings. The interpercentile range (IPR) was defined as:

IPR = P70 − P30

We then calculated the asymmetry index (AI) as:

AI = | 5 − (P30 + P70) / 2 |

The IPRAS value required to declare disagreement was then calculated using the constants proposed in the RAND/UCLA manual:

IPRAS = 2.35 + (1.5 × AI)

An indicator was classified as showing disagreement under the IPRAS method if:

IPR > IPRAS

For transparency and reproducibility, percentiles were computed using standard software percentile functions with linear interpolation (e.g., Excel PERCENTILE.INC for P30 and P70).

We re-classified indicators using the same RAND/UCLA rules (Appropriate: median 7–9 without disagreement; Uncertain: median 4–6 or any disagreement; Inappropriate: median 1–3 without disagreement) and compared results with the primary count-based definition; overall classifications were unchanged.

Supplementary Table S1. Sensitivity analysis of agreement/disagreement classification: count based approach vs IPRAS-based approach (Round 2)

| No. | Quality Indicator | Median (Round2) | 2018 consensus decision | Count-based approach | | | | IPRAS-based approach | | | | |
| --- | --- | --- | --- | --- | --- | --- | --- | --- | --- | --- | --- | --- |
|  |  |  |  | Ratings distribution, n | | | Classification | Percentiles | | | | Classification |
|  |  |  |  | n(9-7) | n(6-4) | n(3-1) | Agreement/ Disagreement | P30 | P70 | IPR | IPRAS | Agreement/ Disagreement |
| 1 | ICU mortality | 9 | Selected | 14 | 0 | 0 | Agreement | 9 | 9 | 0 | 8.35 | Agreement |
| 2 | Hospital mortality | 9 | Selected | 14 | 0 | 0 | Agreement | 8 | 9 | 1 | 7.6 | Agreement |
| 3 | 30 day mortality | 7.5 | Selected | 11 | 3 | 0 | Agreement | 7 | 8 | 1 | 6.1 | Agreement |
| 4 | Standardized mortality rate | 9 | Selected | 14 | 0 | 0 | Agreement | 8 | 9 | 1 | 7.6 | Agreement |
| 5 | Readmission to the ICU | 8 | Selected | 13 | 1 | 0 | Agreement | 7 | 8 | 1 | 6.1 | Agreement |
| 6 | ICU bed occupancy rate | 7 | Selected | 11 | 3 | 0 | Agreement | 7 | 8 | 1 | 6.1 | Agreement |
| 7 | ICU stay lasting 7 days or more | 7 | Selected | 11 | 3 | 0 | Agreement | 7 | 7.1 | 0.1 | 5.43 | Agreement |
| 8 | Length of stay in ICU | 7 | Selected | 12 | 2 | 0 | Agreement | 7 | 8 | 1 | 6.1 | Agreement |
| 9 | Targeted temperature management after cardiac arrest | 6 | Deleted | 3 | 10 | 1 | Agreement | 5 | 6 | 1 | 3.1 | Agreement |
| 10 | Monitoring sedation | 9 | Selected | 14 | 0 | 0 | Agreement | 8 | 9 | 1 | 7.6 | Agreement |
| 11 | Monitoring pain | 9 | Selected | 14 | 0 | 0 | Agreement | 8 | 9 | 1 | 7.6 | Agreement |
| 12 | Identification of delirium | 9 | Selected | 14 | 0 | 0 | Agreement | 8 | 9 | 1 | 7.6 | Agreement |
| 13 | Reintubation | 7 | Selected | 10 | 4 | 0 | Agreement | 6.9 | 8 | 1.1 | 6.03 | Agreement |
| 14 | Duration of mechanical ventilation | 8 | Selected | 13 | 1 | 0 | Agreement | 7.9 | 8 | 0.1 | 6.78 | Agreement |
| 15 | Prolonged mechanical ventilation | 7.5 | Selected | 14 | 0 | 0 | Agreement | 7 | 8 | 1 | 6.1 | Agreement |
| 16 | Postoperative respiratory failure | 7.5 | Selected | 12 | 1 | 1 | Agreement | 7 | 8 | 1 | 6.1 | Agreement |
| 17 | Unplanned extubation | 7 | Selected | 12 | 1 | 1 | Agreement | 7 | 8 | 1 | 6.1 | Agreement |
| 18 | Use of a weaning protocol | 8 | Selected | 13 | 1 | 0 | Agreement | 8 | 8.1 | 0.1 | 6.93 | Agreement |
| 19 | Ventilator-associated pneumonia (VAP) prevention bundle | 8 | Selected | 14 | 0 | 0 | Agreement | 8 | 8 | 0 | 6.85 | Agreement |
| 20 | Low tidal volume ventilation in ARDS | 7.5 | Selected | 12 | 2 | 0 | Agreement | 7 | 8 | 1 | 6.1 | Agreement |
| 21 | Early enteral nutrition | 7.5 | Selected | 11 | 3 | 0 | Agreement | 7 | 8 | 1 | 6.1 | Agreement |
| 22 | Stress ulcer prophylaxis | 6 | Deleted | 4 | 10 | 0 | Agreement | 6 | 6.1 | 0.1 | 3.93 | Agreement |
| 23 | Stress ulcer prophylaxis during mechanical ventilation | 7 | Selected | 10 | 4 | 0 | Agreement | 6.9 | 7.1 | 0.2 | 5.35 | Agreement |
| 24 | Glycemic control | 7 | Selected | 10 | 4 | 0 | Agreement | 7 | 7 | 0 | 5.35 | Agreement |
| 25 | Blood culture sampling | 8 | Selected | 12 | 2 | 0 | Agreement | 7 | 8.1 | 1.1 | 6.18 | Agreement |
| 26 | New-onset MRSA | 7 | Selected | 11 | 3 | 0 | Agreement | 7 | 8 | 1 | 6.1 | Agreement |
| 27 | New-onset multidrug-resistant organisms | 7 | Selected | 12 | 2 | 0 | Agreement | 7 | 7 | 0 | 5.35 | Agreement |
| 28 | Restrictive transfusion strategy in sepsis | 6.5 | Selected | 7 | 7 | 0 | Agreement | 6 | 7 | 1 | 4.6 | Agreement |
| 29 | Venous thromboembolism (VTE) prophylaxis | 8 | Selected | 14 | 0 | 0 | Agreement | 7 | 8 | 1 | 6.1 | Agreement |
| 30 | Hand hygiene | 8 | Selected | 13 | 1 | 0 | Agreement | 8 | 9 | 1 | 7.6 | Agreement |
| 31 | Early rehabilitation | 8 | Selected | 14 | 0 | 0 | Agreement | 7.9 | 8.1 | 0.2 | 6.85 | Agreement |
| 32 | Dedicated intensivist hours | 8 | Selected | 14 | 0 | 0 | Agreement | 8 | 8 | 0 | 6.85 | Agreement |
| 33 | Patient-to-nurse ratio | 8 | Selected | 13 | 1 | 0 | Agreement | 7.9 | 8 | 0.1 | 6.78 | Agreement |
| 34 | Multidisciplinary rounds and daily goal setting | 8 | Selected | 14 | 0 | 0 | Agreement | 7.9 | 8 | 0.1 | 6.78 | Agreement |
| 35 | Standardized handoff communication | 7.5 | Selected | 13 | 1 | 0 | Agreement | 7 | 8 | 1 | 6.1 | Agreement |
| 36 | Adverse event reporting system | 7.5 | Selected | 12 | 2 | 0 | Agreement | 7 | 9 | 2 | 6.85 | Agreement |
| 37 | Incident reporting by physicians | 7.5 | Selected | 13 | 1 | 0 | Agreement | 7 | 8 | 1 | 6.1 | Agreement |
| 38 | Ventilator-associated pneumonia (VAP) per 1,000 ventilator-days | 8 | Selected | 12 | 2 | 0 | Agreement | 8 | 8 | 0 | 6.85 | Agreement |
| 39 | Number of clinical engineers | 7 | Selected | 10 | 4 | 0 | Agreement | 6.9 | 8 | 1.1 | 6.03 | Agreement |
| 40 | Number of pharmacists | 7 | Selected | 11 | 3 | 0 | Agreement | 7 | 8 | 1 | 6.1 | Agreement |

ICU: Intensive Care Unit, VAP: Ventilator-associated pneumonia, ARDS: Acute Respiratory Distress Syndrome, MRSA: Methicillin-Resistant Staphylococcus Aureus

RBC: Red Blood Cell, VTE: Venous Thromboembolism

Supplementary Table S2. Sensitivity analysis of agreement/disagreement classification: count based approach vs IPRAS-based approach (Round 3)

| No. | Quality Indicator | Median (Round3) | 2025 updated  decision | Count-based approach | | | | IPRAS-based approach | | | | |
| --- | --- | --- | --- | --- | --- | --- | --- | --- | --- | --- | --- | --- |
|  |  |  |  | Ratings distribution, n | | | Classification | Percentiles | | | | Classification |
|  |  |  |  | n(9-7) | n(6-4) | n(3-1) | Agreement/ Disagreement | P30 | P70 | IPR | IPRAS | Agreement/ Disagreement |
| 1 | ICU mortality | 9 | Retained | 14 | 0 | 0 | Agreement | 9 | 9 | 0 | 8.35 | Agreement |
| 2 | Hospital mortality | 9 | Retained | 14 | 0 | 0 | Agreement | 8 | 9 | 1 | 7.6 | Agreement |
| 3 | 30 day mortality | 8 | Retained | 12 | 1 | 1 | Agreement | 7 | 8.1 | 1.1 | 6.18 | Agreement |
| 4 | Standardized mortality rate | 9 | Retained | 13 | 1 | 0 | Agreement | 8 | 9 | 1 | 7.6 | Agreement |
| 5 | Readmission to the ICU | 8 | Retained | 13 | 1 | 0 | Agreement | 7.9 | 8.1 | 0.2 | 6.85 | Agreement |
| 6 | ICU bed occupancy rate | 7 | Retained | 11 | 3 | 0 | Agreement | 7 | 8 | 1 | 6.1 | Agreement |
| 7 | ICU stay lasting 7 days or more | 7 | Retained | 11 | 3 | 0 | Agreement | 7 | 7 | 0 | 5.35 | Agreement |
| 8 | Length of stay in ICU | 8 | Retained | 13 | 1 | 0 | Agreement | 7 | 8 | 1 | 6.1 | Agreement |
| 9 | Monitoring sedation | 9 | Retained | 12 | 2 | 0 | Agreement | 9 | 9 | 0 | 8.35 | Agreement |
| 10 | Monitoring pain | 9 | Retained | 13 | 1 | 0 | Agreement | 9 | 9 | 0 | 8.35 | Agreement |
| 11 | Identification of delirium | 9 | Retained | 13 | 1 | 0 | Agreement | 9 | 9 | 0 | 8.35 | Agreement |
| 12 | Reintubation | 7 | Retained | 11 | 3 | 0 | Agreement | 7 | 8 | 1 | 6.1 | Agreement |
| 13 | Duration of mechanical ventilation | 8 | Retained | 13 | 1 | 0 | Agreement | 7 | 8 | 1 | 6.1 | Agreement |
| 14 | Prolonged mechanical ventilation | 8 | Retained | 14 | 0 | 0 | Agreement | 7 | 8 | 1 | 6.1 | Agreement |
| 15 | Postoperative respiratory failure | 7.5 | Retained | 13 | 1 | 0 | Agreement | 7 | 8 | 1 | 6.1 | Agreement |
| 16 | Unplanned extubation | 7.5 | Retained | 11 | 3 | 0 | Agreement | 7 | 8 | 1 | 6.1 | Agreement |
| 17 | Use of a weaning protocol | 8.5 | Retained | 14 | 0 | 0 | Agreement | 8 | 9 | 1 | 7.6 | Agreement |
| 18 | Ventilator-associated pneumonia (VAP)  prevention bundle | 8 | Retained | 14 | 0 | 0 | Agreement | 8 | 9 | 1 | 7.6 | Agreement |
| 19 | Ventilator-associated pneumonia (VAP) per  1,000 ventilator-days | 8 | Retained | 13 | 1 | 0 | Agreement | 8 | 8 | 0 | 6.85 | Agreement |
| 20 | Low tidal volume ventilation in ARDS | 8 | Retained | 13 | 0 | 1 | Agreement | 7 | 8 | 1 | 6.1 | Agreement |
| 21 | Early enteral nutrition | 8 | Retained | 12 | 2 | 0 | Agreement | 7.9 | 9 | 1.1 | 7.53 | Agreement |
| 22 | Stress ulcer prophylaxis during mechanical ventilation | 8 | Retained | 11 | 2 | 1 | Agreement | 7 | 8 | 1 | 6.1 | Agreement |
| 23 | Glycemic control | 8 | Retained | 12 | 1 | 1 | Agreement | 7 | 8 | 1 | 6.1 | Agreement |
| 24 | Blood culture sampling | 8 | Retained | 12 | 1 | 1 | Agreement | 7 | 9 | 2 | 6.85 | Agreement |
| 25 | New-onset MRSA | 7 | Retained | 12 | 2 | 0 | Agreement | 7 | 8 | 1 | 6.1 | Agreement |
| 26 | New-onset multidrug-resistant organisms | 7.5 | Retained | 13 | 1 | 0 | Agreement | 7 | 8 | 1 | 6.1 | Agreement |
| 27 | Restrictive transfusion strategy in sepsis | 6 | Not Retained | 6 | 7 | 1 | Agreement | 6 | 7 | 1 | 4.6 | Agreement |
| 28 | Venous thromboembolism (VTE)  prophylaxis | 8 | Retained | 12 | 2 | 0 | Agreement | 8 | 8 | 0 | 6.85 | Agreement |
| 29 | Hand hygiene | 8 | Retained | 13 | 0 | 1 | Agreement | 8 | 9 | 1 | 7.6 | Agreement |
| 30 | Early rehabilitation | 8 | Retained | 14 | 0 | 0 | Agreement | 8 | 9 | 1 | 7.6 | Agreement |
| 31 | Dedicated intensivist hours | 8 | Retained | 13 | 0 | 1 | Agreement | 8 | 8.1 | 0.1 | 6.93 | Agreement |
| 32 | Patient-to-nurse ratio | 8 | Retained | 13 | 1 | 0 | Agreement | 8 | 8.1 | 0.1 | 6.93 | Agreement |
| 33 | Multidisciplinary rounds and daily goal  setting | 9 | Retained | 14 | 0 | 0 | Agreement | 8 | 9 | 1 | 7.6 | Agreement |
| 34 | Standardized handoff communication | 8 | Retained | 13 | 1 | 0 | Agreement | 7 | 8 | 1 | 6.1 | Agreement |
| 35 | Adverse event reporting system | 8 | Retained | 14 | 0 | 0 | Agreement | 7.9 | 8.1 | 0.2 | 6.85 | Agreement |
| 36 | Incident reporting by physicians | 8 | Retained | 14 | 0 | 0 | Agreement | 7.9 | 8.1 | 0.2 | 6.85 | Agreement |
| 37 | Number of clinical engineers | 8 | Retained | 12 | 2 | 0 | Agreement | 7.9 | 8 | 0.1 | 6.78 | Agreement |
| 38 | Number of pharmacists | 8 | Retained | 12 | 2 | 0 | Agreement | 7 | 8 | 1 | 6.1 | Agreement |
| 39 | Ventilator-associated events (VAE) per 1,000  ventilator-days | 8 | Added | 14 | 0 | 0 | Agreement | 7 | 8 | 1 | 6.1 | Agreement |

ICU: Intensive Care Unit, VAP: Ventilator-associated pneumonia, ARDS: Acute Respiratory Distress Syndrome, MRSA: Methicillin-Resistant Staphylococcus Aureus

RBC: Red Blood Cell, VTE: Venous Thromboembolism, VAE: Ventilator-associated events

Supplementary Table S3. Mapping of the Japanese ICU Quality Indicators with International Frameworks and Data Source Feasibility

| No. | Japanese ICU QIs | Donabedian Category | 2025 updated decision | Correspondence with Global Standards | Japan Specific Context | Data Sources |
| --- | --- | --- | --- | --- | --- | --- |
| 1 | ICU mortality | Outcome | Retained | AHRQ (ii) | Internationally common;  applicable in Japan | JIPAD, Administrative claims data |
| 2 | Hospital mortality | Outcome | Retained | ACHS (ii)  AHRQ (ii) | Internationally common;  applicable in Japan | JIPAD, Administrative claims data |
| 3 | 30 day mortality | Outcome | Retained | AHRQ (ii) | Internationally common;  applicable in Japan | JIPAD, Administrative claims data |
| 4 | Standardized mortality rate | Outcome | Retained | ESICM (i)  SICSAG (i)  NICE (i)  AHRQ (i) | Internationally common;  applicable in Japan | JIPAD |
| 5 | Readmission to the ICU | Outcome | Retained | ESICM (i) | Internationally common;  applicable in Japan | JIPAD, Administrative claims data |
| 6 | ICU bed occupancy rate | Structure | Retained | NICE (ii) | Internationally common;  applicable in Japan | JIPAD, Administrative claims data, Survey data |
| 7 | ICU stay lasting 7 days or more | Outcome | Retained | Not included as a formal  indicator in the mapped  frameworks | Not explicitly listed in mapped QI sets; local benchmarking choice | JIPAD, Administrative claims data |
| 8 | Length of stay in ICU | Outcome | Retained | NICE (i) | Internationally common;  applicable in Japan | JIPAD, Administrative claims data |
| 9 | Monitoring sedation | Structure | Retained | DIVI (ii)  AHRQ (ii) | Internationally common;  applicable in Japan | Survey data |
| 10 | Monitoring pain | Structure | Retained | DIVI (ii)  AHRQ (ii) | Internationally common;  applicable in Japan | Survey data |
| 11 | Identification of delirium | Structure | Retained | DIVI (ii)  AHRQ (ii) | Internationally common;  applicable in Japan | Survey data |
| 12 | Reintubation | Outcome | Retained | Not included as a formal  indicator in the mapped  frameworks | Common performance metric; not formalized in mapped ICU QI sets | JIPAD, Administrative claims data |
| 13 | Duration of mechanical ventilation | Outcome | Retained | NICE (i) | Internationally common;  applicable in Japan | JIPAD, Administrative claims data |
| 14 | Prolonged mechanical ventilation | Outcome | Retained | Not included as a formal  indicator in the mapped  frameworks | Definition varies; not formalized in mapped ICU QI sets | JIPAD, Administrative claims data |
| 15 | Postoperative respiratory failure | Outcome | Retained | Not included as a formal  indicator in the mapped  frameworks | Perioperative outcome metric; not formalized in mapped ICU QI sets | JIPAD |
| 16 | Unplanned extubation | Outcome | Retained | ESICM (i)  NICE (i) | Internationally common;  applicable in Japan | Electronic health records |
| 17 | Use of a weaning protocol | Structure | Retained | DIVI (ii) | Internationally common;  applicable in Japan | Survey data |
| 18 | Ventilator-associated pneumonia (VAP)  prevention bundle | Process | Retained | SICSAG (i) | Internationally common;  applicable in Japan | Electronic health records |
| 19 | Ventilator-associated pneumonia (VAP)  per 1,000 ventilator-days | Outcome | Retained | US CDC/NHSN (i)  AHRQ (i)  WHO-domain (iii) | Internationally common;  applicable in Japan | Electronic health records |
| 20 | Low tidal volume ventilation in ARDS | Process | Retained | Not included as a formal  indicator in the mapped  frameworks | Guideline-driven practice;  feasibility concerns | Electronic health records |
| 21 | Early enteral nutrition | Process | Retained | AHRQ (ii) | Internationally common;  applicable in Japan | Administrative claims data |
| 22 | Stress ulcer prophylaxis during  mechanical ventilation | Process | Retained | AHRQ (ii) | Internationally common;  applicable in Japan | Administrative claims data |
| 23 | Glycemic control | Process | Retained | NICE (ii) | Internationally common;  applicable in Japan | Laboratory data |
| 24 | Blood culture sampling | Process | Retained | WHO-domain (iii) | Internationally common;  applicable in Japan | Administrative claims data |
| 25 | New-onset MRSA | Outcome | Retained | WHO-domain (iii) | Internationally common;  applicable in Japan | Electronic health records |
| 26 | New-onset multidrug-resistant organisms | Outcome | Retained | WHO-domain (iii) | Internationally common;  applicable in Japan | Electronic health records |
| 27 | Restrictive transfusion strategy in sepsis | Process | Not Retained | Not included as a formal  indicator in the mapped  frameworks | Guideline-driven practice;  feasibility concerns | Electronic health records |
| 28 | Venous thromboembolism (VTE)  prophylaxis | Process | Retained | ACHS (i)  AHRQ (i)  WHO-domain (iii) | Internationally common;  applicable in Japan | Administrative claims data |
| 29 | Hand hygiene | Process | Retained | AHRQ (ii)  WHO-domain (iii) | Internationally common;  applicable in Japan | Facility-reported results from locally audited monitoring |
| 30 | Early rehabilitation | Process | Retained | SICSAG (i)  DIVI (ii) | Internationally common;  applicable in Japan | Administrative claims data |
| 31 | Dedicated intensivist hours | Structure | Retained | ESICM (ii)  ACHS (ii)  NICE (ii) | Internationally common;  applicable in Japan | Survey data |
| 32 | Patient-to-nurse ratio | Structure | Retained | ACHS (ii)  NICE (ii) | Internationally common;  applicable in Japan | Survey data |
| 33 | Multidisciplinary rounds and daily goal  setting | Structure | Retained | ESICM (i)  DIVI (ii) | Internationally common;  applicable in Japan | Survey data |
| 34 | Standardized handoff communication | Structure | Retained | ESICM (i) | Internationally common;  applicable in Japan | Survey data |
| 35 | Adverse event reporting system | Structure | Retained | ESICM (i)  NICE (ii) | Internationally common;  applicable in Japan | Survey data |
| 36 | Incident reporting by physicians | Structure | Retained | Not included as a formal  indicator in the mapped  frameworks | Reflects Japan-specific  staffing/regulatory context | Survey data |
| 37 | Number of clinical engineers | Structure | Retained | Not included as a formal  indicator in the mapped  frameworks | Reflects Japan-specific  staffing/regulatory context | Survey data |
| 38 | Number of pharmacists | Structure | Retained | Not included as a formal  indicator in the mapped  frameworks | Reflects Japan-specific  staffing/regulatory context | Survey data |
| 39 | Ventilator-associated events (VAE)  per 1,000 ventilator-days | Outcome | Added | US CDC/NHSN (i)  WHO-domain (iii) | Internationally common;  applicable in Japan | Electronic health records |

Notes:

The “Correspondence with Global Standards” column classifies the alignment as follows: (i) the indicator definition is essentially the same; (ii) a closely related indicator is specified but definitions may differ; (iii) alignment at the broader domain level (e.g., WHO patient safety/IPC/AMR).

In this study, “Survey data” refer to institutional (facility-level) surveys (e.g., staffing, protocols, and safety systems) rather than patient-reported surveys.

Global standard sources used for mapping include: ESICM, SICSAG, DIVI, ACHS, NICE, CDC/NHSN, AHRQ, WHO-domain

Abbreviations: QI: quality indicator, ESICM: European Society of Intensive Care Medicine, SICSAG: Scottish Intensive Care Society Audit Group, DIVI: German Deutsche Interdisziplinäre Vereinigung für Intensiv- und Notfallmedizin, ACHS: Australian Council on Healthcare Standards, NICE (Dutch): National Intensive Care Evaluation (Netherlands), US CDC/NHSN: United State Center for Disease Control/National Healthcare Safety Network, AHRQ: Agency for Healthcare Research and Quality (US), WHO: World Health Organization, JIPAD: Japanese Intensive care PAtient Database, VAP: Ventilator-associated pneumonia, VAE: Ventilator-associated events, VTE: Venous thromboembolism

Supplementary Table S4. Comparison of appropriateness ratings between Round 2 (2018) and Round 3 (2025)

| No. | Quality Indicator | Round 2 (2018) | | | | Round 3 (2025) | | | |
| --- | --- | --- | --- | --- | --- | --- | --- | --- | --- |
|  |  | Median | Ratings distribution, n | | | Median | Ratings distribution, n | | |
|  |  |  | n(9-7) | n(6-4) | n(3-1) |  | n(9-7) | n(6-4) | n(3-1) |
| 1 | ICU mortality | 9 | 14 | 0 | 0 | 9 | 14 | 0 | 0 |
| 2 | Hospital mortality | 9 | 14 | 0 | 0 | 9 | 14 | 0 | 0 |
| 3 | 30 day mortality | 7.5 | 11 | 3 | 0 | 8 | 12 | 1 | 1 |
| 4 | Standardized mortality rate | 9 | 14 | 0 | 0 | 9 | 13 | 1 | 0 |
| 5 | Readmission to the ICU | 8 | 13 | 1 | 0 | 8 | 13 | 1 | 0 |
| 6 | ICU bed occupancy rate | 7 | 11 | 3 | 0 | 7 | 11 | 3 | 0 |
| 7 | ICU stay lasting 7 days or more | 7 | 11 | 3 | 0 | 7 | 11 | 3 | 0 |
| 8 | Length of stay in ICU | 7 | 12 | 2 | 0 | 8 | 13 | 1 | 0 |
| 9 | Monitoring sedation | 9 | 14 | 0 | 0 | 9 | 12 | 2 | 0 |
| 10 | Monitoring pain | 9 | 14 | 0 | 0 | 9 | 13 | 1 | 0 |
| 11 | Identification of delirium | 9 | 14 | 0 | 0 | 9 | 13 | 1 | 0 |
| 12 | Reintubation | 7 | 10 | 4 | 0 | 7 | 11 | 3 | 0 |
| 13 | Duration of mechanical ventilation | 8 | 13 | 1 | 0 | 8 | 13 | 1 | 0 |
| 14 | Prolonged mechanical ventilation | 7.5 | 14 | 0 | 0 | 8 | 14 | 0 | 0 |
| 15 | Postoperative respiratory failure | 7.5 | 12 | 1 | 1 | 7.5 | 13 | 1 | 0 |
| 16 | Unplanned extubation | 7 | 12 | 1 | 1 | 7.5 | 11 | 3 | 0 |
| 17 | Use of a weaning protocol | 8 | 13 | 1 | 0 | 8.5 | 14 | 0 | 0 |
| 18 | Ventilator-associated pneumonia (VAP) prevention bundle | 8 | 14 | 0 | 0 | 8 | 14 | 0 | 0 |
| 19 | Ventilator-associated pneumonia (VAP) per 1,000 ventilator-days | 8 | 12 | 2 | 0 | 8 | 13 | 1 | 0 |
| 20 | Low tidal volume ventilation in ARDS | 7.5 | 12 | 2 | 0 | 8 | 13 | 0 | 1 |
| 21 | Early enteral nutrition | 7.5 | 11 | 3 | 0 | 8 | 12 | 2 | 0 |
| 22 | Stress ulcer prophylaxis during mechanical ventilation | 7 | 10 | 4 | 0 | 8 | 11 | 2 | 1 |
| 23 | Glycemic control | 7 | 10 | 4 | 0 | 8 | 12 | 1 | 1 |
| 24 | Blood culture sampling | 8 | 12 | 2 | 0 | 8 | 12 | 1 | 1 |
| 25 | New-onset MRSA | 7 | 11 | 3 | 0 | 7 | 12 | 2 | 0 |
| 26 | New-onset multidrug-resistant organisms | 7 | 12 | 2 | 0 | 7.5 | 13 | 1 | 0 |
| 27 | Restrictive transfusion strategy in sepsis | 6.5 | 7 | 7 | 0 | 6 | 6 | 7 | 1 |
| 28 | Venous thromboembolism (VTE) prophylaxis | 8 | 14 | 0 | 0 | 8 | 12 | 2 | 0 |
| 29 | Hand hygiene | 8 | 13 | 1 | 0 | 8 | 13 | 0 | 1 |
| 30 | Early rehabilitation | 8 | 14 | 0 | 0 | 8 | 14 | 0 | 0 |
| 31 | Dedicated intensivist hours | 8 | 14 | 0 | 0 | 8 | 13 | 0 | 1 |
| 32 | Patient-to-nurse ratio | 8 | 13 | 1 | 0 | 8 | 13 | 1 | 0 |
| 33 | Multidisciplinary rounds and daily goal setting | 8 | 14 | 0 | 0 | 9 | 14 | 0 | 0 |
| 34 | Standardized handoff communication | 7.5 | 13 | 1 | 0 | 8 | 13 | 1 | 0 |
| 35 | Adverse event reporting system | 7.5 | 12 | 2 | 0 | 8 | 14 | 0 | 0 |
| 36 | Incident reporting by physicians | 7.5 | 13 | 1 | 0 | 8 | 14 | 0 | 0 |
| 37 | Number of clinical engineers | 7 | 10 | 4 | 0 | 8 | 12 | 2 | 0 |
| 38 | Number of pharmacists | 7 | 11 | 3 | 0 | 8 | 12 | 2 | 0 |
| 39 | Ventilator-associated events (VAE) per 1,000 ventilator-days | - | - | - | - | 8 | 14 | 0 | 0 |

ICU: Intensive Care Unit, VAP: Ventilator-associated pneumonia, ARDS: Acute Respiratory Distress Syndrome, MRSA: Methicillin-Resistant Staphylococcus Aureus

RBC: Red Blood Cell, VTE: Venous Thromboembolism, VAE: Ventilator-associated events

Footnote. Values are median appropriateness score (9-point Likert scale) and the number of panelists rating each indicator in the 7–9, 4–6, and 1–3 ranges (n=14). Indicator No. 39 was newly proposed and rated only in Round 3.
